# Effect of High Ni Content in Gas-Atomized Cu-Ni-Si Powders for Laser Powder Bed Fusion

**DOI:** 10.3390/ma18204772

**Published:** 2025-10-18

**Authors:** Mirko Trovato, Nicolò Arcieri, Diego Manfredi, Federico Simone Gobber, Bhaskaranand Bhatt, Alessandra Martucci, Sara Biamino, Laura Montanaro, Mariangela Lombardi, Paolo Fino

**Affiliations:** 1 Department of Applied Science and Technology, Politecnico di Torino, Corso Duca Degli Abruzzi 24, 10129 Turin, Italy; nicolo.arcieri@polito.it (N.A.); diego.manfredi@polito.it (D.M.); federico.gobber@polito.it (F.S.G.); bhaskaranand.bhatt@polito.it (B.B.); alessandra.martucci@polito.it (A.M.); sara.biamino@polito.it (S.B.); laura.montanaro@polito.it (L.M.); mariangela.lombardi@polito.it (M.L.); paolo.fino@polito.it (P.F.); 2 Interdepartmental Center of Integrated Additive Manufacturing (IAM@PoliTo), Politecnico di Torino, Corso Castelfidardo 51, 10129 Turin, Italy

**Keywords:** Cu-Ni-Si alloys, rapid solidification, gas-atomization, powder characterization

## Abstract

Cu-Ni-Si alloys are advanced materials for electronic applications combining high mechanical strength and electrical conductivity through precipitation of fine Ni silicides. Increasing the Ni content—and, thus, the Ni:Si ratio—enhances the volume fraction of strengthening precipitates. However, the conventional fabrication route is time-consuming and costly, as the slow cooling rates lead to a coarse microstructure and pronounced segregation, limiting Ni and Si content to 5 wt.%. Rapid solidification techniques offer a promising alternative, since the higher cooling rates refine the microstructure while suppressing the elemental segregation. This study presents a novel powder-based approach to overcome the compositional limitations of Cu-Ni-Si alloys, providing a pathway for faster alloy screening. Two gas-atomized powders with different Ni contents—CuNi3Si1.5 and CuNi10Si1.5 (wt.%)—were engineered as feedstock for laser powder bed fusion, produced, and characterized to assess the effect of the Ni level on the microstructure and properties. Gas-atomization yielded spherical powders with a fine dendritic structure and limited segregation. Increased Ni content enhanced strengthening mechanisms and hardness, as well as improved optical response, suggesting the potential of high-Ni Cu-Ni-Si compositions for use in laser powder bed fusion.

## 1. Introduction

Cu-Ni-Si alloys, also known as Corson alloys, are a class of precipitation-hardenable (PH) Cu alloys that show a remarkable combination of mechanical and electrical properties, thus making them highly suitable for use in the lead frames of large-scale integrated circuits, high-speed rail transit, optoelectronic devices, and the electronics industry [1]. The precipitation of strengthening intermetallic phases, such as Ni silicides—particularly δ-Ni_2_Si—is responsible for the superior properties of these advanced materials, which are good candidates as substitutes for beryllium-containing alloys, known to be toxic to human health [2]. Indeed, the formation of evenly distributed nanostructured precipitates during aging enhances the mechanical properties via precipitation hardening and reduces the electrical resistivity by depleting the matrix from solute atoms [3]. In this context, the strength of this class of Cu alloys could be enhanced with additional alloying elements, like Cr, Zr, Al, or Mg, or by increasing the Ni:Si ratio, as these strategies promote the formation of a higher volume fraction of strengthening precipitates [4]. However, conventional fabrication of Cu-Ni-Si alloys is a time-consuming and expensive thermo-mechanical route, which involves casting followed by homogenization and solution heat treatment, cold rolling, and, finally, aging [5]. Such a multi-step fabrication strategy is essential, as the slow cooling rate leads to a coarsened microstructure with large precipitates [6]. Moreover, it also promotes the segregation of alloying elements, resulting in the formation of a Ni- and Si-rich network at the boundaries of the primary solidified α-Cu matrix grains—with a face-centered cubic (FCC) structure—via a eutectic reaction in the residual liquid, ultimately degrading both mechanical and electrical properties [7]. Therefore, the thermo-mechanical steps are crucial to break down such a casting-induced reticular structure [8], a phenomenon that typically restricts the total Ni and Si content in current Cu-Ni-Si alloys to 5 wt.% [9].

Powder metallurgy (PM) techniques, as well as rapid solidification (RS) processes, are promising manufacturing approaches to overcome the issues related to the traditional fabrication route of Cu-Ni-Si alloys. In PM, the limited atomic mobility inherent to solid-state processing mitigates the segregation phenomena [8], enabling the development of systems with higher Ni content, aimed at increasing the Ni:Si ratio. For instance, compositions such as CuNi8.33Si1.67 (wt.%) have been successfully developed via PM routes, generally involving mechanical alloying of elemental powders and subsequent consolidation through press and sinter [10,11]. RS methods, on the other hand, achieve cooling rates several orders of magnitude higher than conventional casting. Typical values range from 10^4^ to 10^6^ K·s^−1^, compared to 10^0^–10^2^ K·s^−1^ for conventional solidification [12]. Such high cooling rates effectively suppress elemental segregation—beneficial for electrical conductivity—while promoting, at the same time, grain refinement and formation of nanoscale precipitates, both contributing to enhanced mechanical properties [13,14]. In this context, laser-beam powder bed fusion of metals (PBF-LB/M) represents a particularly promising solution, as it combines the benefit of RS with the advantages of additive manufacturing (AM), such as rapidity and flexibility, as there is no need for molds and tools, capability to fabricate lightweight and intricate components, and efficient use of raw materials [15,16]. However, the literature on Cu-Ni-Si alloys processed via PBF-LB/M is still limited, primarily because of the challenging processability of Cu alloys. Indeed, their typical high thermal conductivity exacerbates the thermal gradient, with detrimental consequences for the process and the part quality [17]. Furthermore, Cu alloys show a very high reflectivity (~95% at room temperature) if irradiated with near-infrared laser, making it more challenging to obtain fully dense components [18]. The low availability of these alloys on the market as powder feedstock may also represent a contributing factor. Nevertheless, the existing works indicate that these systems may offer valuable processing- and property-related benefits. PBF-LB/M-processed CuNi1.5Si and CuNi3Si, particularly the latter, exhibited superior mechanical properties after heat treatment compared to their conventional counterparts [19]. Ventura et al. [20], in their work, demonstrated that aging can be performed directly, without prior solution heat treatment, for the C70250 alloy (a CuNi3Si alloy with a small amount of Mg), potentially reducing the overall production time and cost in an industrial context. More recently, another investigation on PBF-LB/M CuNi3Si alloys reported promising corrosion performance and demonstrated the effectiveness of hot-isostatic pressing (HIP) as a post-processing treatment. The application of HIP led to improvements in density and hardness, with even more significant enhancements in electrical conductivity [21].

To the best of the authors’ knowledge, however, most research efforts on rapidly solidified Cu-Ni-Si alloys remain confined to standard compositions, typically containing only minor additions of Ni and Si (e.g., Ni ≤ 3 wt.% and Si < 1 wt.%), and the exploration of Cu-Ni-Si systems with higher Ni:Si ratios remains limited, especially for PBF-LB/M. In this context, the present work aims to provide a comprehensive characterization of two gas-atomized Cu-Ni-Si systems specifically intended for use in PBF-LB/M, focusing on how the increased Ni content—beyond conventional levels—affects the resulting microstructure and properties. This approach is further supported by a previous study on a different system (AlFe18Si8Cr5Ni2), which demonstrated that the alloy chemistry and phase development can persist through the transition from powder to solidified material during PBF-LB/M, owing to the high cooling rates of both gas-atomization and the AM process [22]. Although thermal cycling during PBF-LB/M affects phase stability and growth, a thorough characterization of the starting powder provides valuable insights into the compositional and microstructural features, ultimately supporting the screening of new systems.

## 2. Materials and Methods

Powders for the two Cu-Ni-Si systems, CuNi3Si1.5 and CuNi10Si1.5 (wt.%), were produced using a vacuum-induction melting, inert-gas-atomization (VIGA) process with close-coupled nozzles on a Hermiga 100/10 atomizer (Phoenix Scientific Industries Ltd., Hailsham, United Kindom). For both runs, pure elemental raw materials—Cu-ETP (>99.90 wt.%, Musola Metalli S.p.A., San Martino Buon Albergo, Italy), Ni (>99.98 wt.%, A.M.P.E.R.E. Alloys, Saint-Ouen-l’Aumône, France), and Si (>99.17 wt.%, A.M.P.E.R.E. Alloys, Saint-Ouen-l’Aumône, France)—were used. Each batch consisted of a total charge of 8 kg. The nominal compositions (wt.%) and the corresponding weight of each raw element are listed in Table 1. Compared to the conventional CuNi3Si alloy, the minor alloyed CuNi3Si1.5 shows a slightly higher Si content, which is considered to be beneficial for PBF-LB/M processability, since it reduces the surface tension of Cu [23]. In order to evaluate only the effect of the Ni increase, the Si content was also maintained at the same level in the CuNi10Si1.5 system.

The raw materials were heated in an alumina crucible, having previously evacuated both the melting and atomization chambers down to 5 Pa. Before the melting, the two chambers were filled with Ar as a shielding gas, with an overpressure of 2 kPa in the melting chamber. When the loaded material reached the molten state, the overpressure in the melting chamber was increased in order to facilitate and to stabilize the melt flow through the nozzle, and the atomization occurred by impinging the molten-metal stream with a high-pressure flow of Ar. The atomization of the CuNi3Si1.5 system was carried out with an overpressure of 24 ± 4 kPa, die pressure of 4.2 ± 0.2 MPa, and melt bath temperature of 1463 °C. Regarding the CuNi10Si1.5 system, the atomization was carried out with an overpressure of 22 ± 2 kPa barg and a die pressure of 4.3 ± 0.1 MPa, as well as a melt bath temperature of 1475 °C.

The powders were then collected and sieved to obtain a fraction with a particle size distribution (PSD) of 20–63 μm (typically employed in PBF-LB/M processes) using a Retsch AS 200 basic vibratory sieve shaker (Retsch GmbH, Haan, Germany) with 200 mm diameter sieves. The chemical compositions of the two batches were assessed by iCAP 7200 ICP-OES (Thermo Fisher Scientific, Waltham, MA, USA), while the light element contents were evaluated, on three samples, with the inert-gas fusion technique (ONH 836, LECO Corporation, St. Joseph, MI, USA).

X-ray diffraction (XRD) analyses were performed for phase identification and to evaluate the lattice parameters. An Empyrean X-ray diffractometer (Malvern Panalytical, Almelo, The Netherlands) was employed in Bragg–Brentano configuration with Cu Kα radiation (*λ*_Cu_ = 1.5406 Å) of 40 kV and 40 mA. Diffraction data were collected in the diffraction angle range of 20° ≤ 2*θ* ≤ 140°, with a step size of 0.003° and sampling time of 180 s per step. The collected patterns were analyzed with HighScore Plus software (version 3.0e, Malvern Panalytical, Almelo, The Netherlands) for phase identification. Lattice parameters were evaluated with the cos*θ*cot*θ* method, which relies on linear extrapolation of the lattice parameter as a function of cos^2^*θ*cot*θ* to obtain a value at *θ* = 90°, thereby reducing systematic errors (e.g., specimen displacement and transparency) [24].

The PSD in numbers was measured through static image analysis with Morphologi 4 (Malvern Panalytical, Malvern, United Kingdom), which also provides information about the shape factors. Each measurement involved the analysis of tens of thousands of particles, ensuring statistical reliability. In particular, the circularity and aspect ratio were calculated according to Equations (1) and (2), respectively, where *A* is the projected area of the particle, *P* is the perimeter of the particle, *d_min_* is the minimum Feret diameter, and *d_max_* is the maximum Feret diameter [25]. Both parameters are dimensionless shape factors.

(1) $$C=\frac{4\pi A}{P^{2}},$$

(2) $$A_{R}=\frac{d_{min}}{d_{max}}.$$

Furthermore, a morphological investigation of the powders on stubs was carried out by means of a Zeiss EVO 15 scanning electron microscope (SEM, Carl Zeiss AG, Oberkochen, Germany) operating at 20 kV voltage with secondary electron (SE) detector.

A microstructural investigation of the two atomized systems was carried out using a Phenom ProX scanning electron microscope (SEM, Thermo Fisher Scientific, Eindhoven, The Netherlands) equipped with energy-dispersive X-ray analysis (EDX), operating at a 15 kV voltage with a back-scattered electron (BSE) detector. The two powders with the different chemical compositions were hot mounted in resin and underwent metallographic preparation, consisting of successive grinding with SiC papers up to 4000 grit, followed by polishing with diamond suspensions down to 1 μm and a final polishing with colloidal silica to achieve a mirror-like finish. EDX analyses were carried out to examine the alloying elements’ distribution. In addition, a field-emission scanning electron microscope (FESEM, Merlin, Carl Zeiss AG, Oberkochen, Germany), operated in the InLens mode, was employed for high-magnification observations. Prior to imaging, the samples were etched to allow for clearer visualization of the microstructural morphology. CuNi3Si1.5 particles were etched with a NH_4_OH (50 mL) + H_2_O_2_ (100 mL) + DI H_2_O (50 mL) solution, while CuNi10Si1.5 ones were etched with an FeCl_3_ (5 g) + HCl (20 mL) + H_2_O (100 mL) solution.

Transmission electron microscopy (TEM, Talos F200X, Thermo Fisher Scientific, Eindhoven, The Netherlands) was employed to determine the distribution of elements and precipitates at the submicron scale using multiple imaging modes and an energy-dispersive X-ray spectrometer (Super-X G2 EDX Detector, Thermo Fisher Scientific, Hillsboro, OR, USA). Thin lamellae (<100 nm) of the powder particles for TEM observations were prepared in three steps, using a focused ion beam (FIB) on a SOLARIS dual-beam system FIB/SEM (TESCAN, Brno-Kohoutovice, Czech Republic). Particles (30 to 45 µm) of both gas-atomized powders were initially mounted to a half-moon TEM molybdenum grid using platinum coating and a manipulator. Then, they were thinned and polished with the ion beam at 3–30 keV to achieve a thickness of less than 100 nm.

Nanoindentation hardness tests were performed in compliance with the ISO 14577 standard [26]. The particles were embedded in resin and underwent metallographic preparation up to a mirror-like finish. A load-controlled method was employed using the TI 950 Triboindenter (Bruker Nano Surfaces, Minneapolis, MN, USA) equipped with a Berkovich tip. The tests were conducted with a peak load of 1000 µN, held for 2 s at maximum load. A total of 100 indentations were arranged in a 10 × 10 matrix, maintaining a 3 µm spacing to prevent plastic deformation overlap between adjacent indentations. The procedure was fully automated, ensuring precise control over sample positioning, loading, holding, unloading, retraction, and movement to subsequent indentation points. Both loading and unloading rates were set at 200 µN/s, with a 2 s hold at peak load before acquiring the load–depth curves. A minimal thermal drift of 0.10 nm/s was maintained to minimize experimental errors. The obtained load–displacement curves were analyzed using dedicated nanoindentation software, and nanohardness values were extracted for analysis. This methodology ensured high accuracy and repeatability in assessing the local mechanical properties of the powder systems under investigation.

Diffuse reflectance spectra of the two gas-atomized alloys, together with pure Cu powder, were measured with the ultraviolet/visible light (UV-Vis) spectrophotometer Varian Cary 5000 (Agilent Technologies, Leinì, Italy) equipped with an integrated sphere in the wavelength range from 400 to 1500 nm and with a wavelength step of 0.66 nm. The corresponding absorption spectra *F*(*R*) were then evaluated according to the Kubelka–Munk theory [27] with the following equation:

(3) $$F\left(R_{\infty }\right)=\frac{K}{S}=\frac{{\left(1-R_{\infty }\right)}^{2}}{2R_{\infty }},$$

where *R_∞_* is the reflectance of an infinitely thick specimen, *K* is the absorption coefficient, and *S* is the scattering coefficient. *F*(*R*) is a dimensionless function proportional to the absorption-to-scattering ratio.

## 3. Results and Discussion

As already stated, the compositional and microstructural characteristics of gas-atomized powders should provide relevant information about the phase development and the potential mechanical properties of PBF-LB/M materials.

The chemical compositions of the two gas-atomized powders are reported in Table 2, together with the light element content. In both systems, the actual Si content is approximately 10% lower than the nominal one, whereas the variations in Ni content are comparatively smaller. Light-element content analysis revealed a modest oxygen content, equal to 0.013 ± 0.002 wt.% and 0.019 ± 0.002 wt.% for CuNi3Si1.5 and CuNi10Si1.5, respectively.

**Figure 1:**
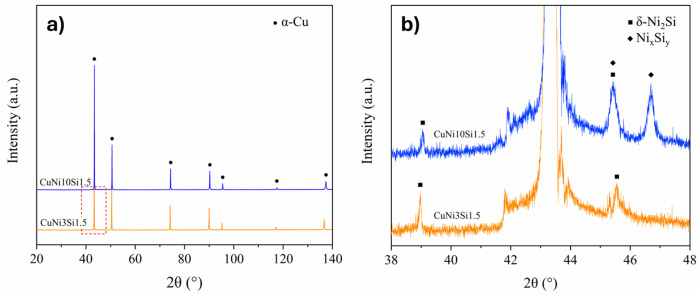
(**a**) XRD pattern of CuNi3Si1.5 (orange line) and CuNi10Si1.5 (blue line); (**b**) magnified view of the range 2θ = 38–48° highlighted by red box in (**a**).

Upon examining the diffraction patterns displayed in Figure 1a, the peaks of the α-Cu solid solution are clearly visible in both gas-atomized compositions. Thus, α-Cu can be interpreted as the matrix phase with a face-centered cubic (FCC) structure. Considering the standard Cu lattice parameter, *a* = 3.6150 Å (PDF card 04-0836), the lattice parameters of *a* = 3.6131 Å and *a* = 3.6042 Å were calculated for CuNi3Si1.5 and CuNi10Si1.5, respectively. The calculated lattice parameters revealed a contraction of the cubic crystal lattice, particularly in the system with higher Ni content. It is worth nothing that Ni and Si have opposite effects on the Cu lattice: Ni tends to contract the lattice, whereas Si promotes its expansion [28]. Therefore, the observed overall contraction reflects the dominant effect of Ni over Si within the Cu matrix of the two gas-atomized powders. A closer inspection of the magnified patterns in Figure 1b shows further diffraction peaks with weak intensities. As regards the CuNi3Si1.5 powders (orange line), these peaks are likely related to the δ-Ni_2_Si phase [29]. Conversely, the diffraction pattern of the CuNi10Si1.5 powders (blue line) suggests the occurrence of an additional Ni_x_Si_y_ phase alongside the δ-Ni_2_Si phase, which cannot be precisely identified. This uncertainty should be primarily attributed to the fine size and low volume fraction of the precipitates.

D10, D50, and D90 number-based percentiles for the PSD, circularity, and aspect ratio of the two Cu-Ni-Si systems are reported in Table 3. Considering the PSD, even though the composition with higher Ni exhibits a slight tendency toward finer particles, a 45% yield in the range 20–63 μm was obtained in both cases. By looking at the percentiles of the shape factors, each batch shows a good overall particle shape, which is beneficial for further processing with PBF-LB/M machines [30].

**Figure 2:**
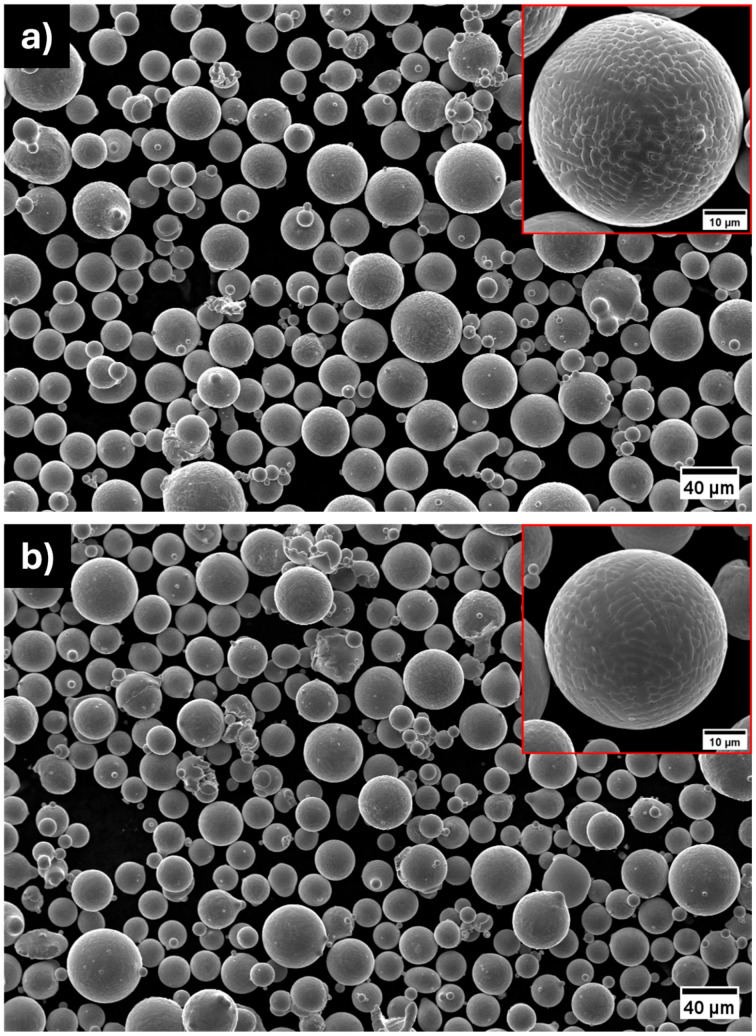
SEM micrographs of gas-atomized (**a**) CuNi3Si1.5 and (**b**) CuNi10Si1.5 powders. Insets (red boxes) show higher-magnification views revealing the fine dendritic solidification structure.

The results obtained from the Morphologi are consistent with the morphological SEM micrographs depicted in Figure 2. Particles from the two gas-atomized batches exhibit a nearly spherical geometry with a low number of defects, namely, satellites, splat caps, and irregular shapes. When observed at higher magnification, particles of both compositions are characterized by a fine dendritic structure, typical of gas-atomized powders due to the rapid solidification of the process [31]. The formation of these morphologies can be described according to constitutional supercooling theory. During the solidification of an alloy, solute elements rejected at the solid–liquid interface accumulate in the adjacent liquid, lowering its liquidus temperature. If the actual thermal gradient is too low relative to the growth rate, a region of supercooled liquid forms ahead of the solidification front. This condition destabilizes the planar interface and promotes the development of cellular or dendritic structures [32].

**Figure 3:**
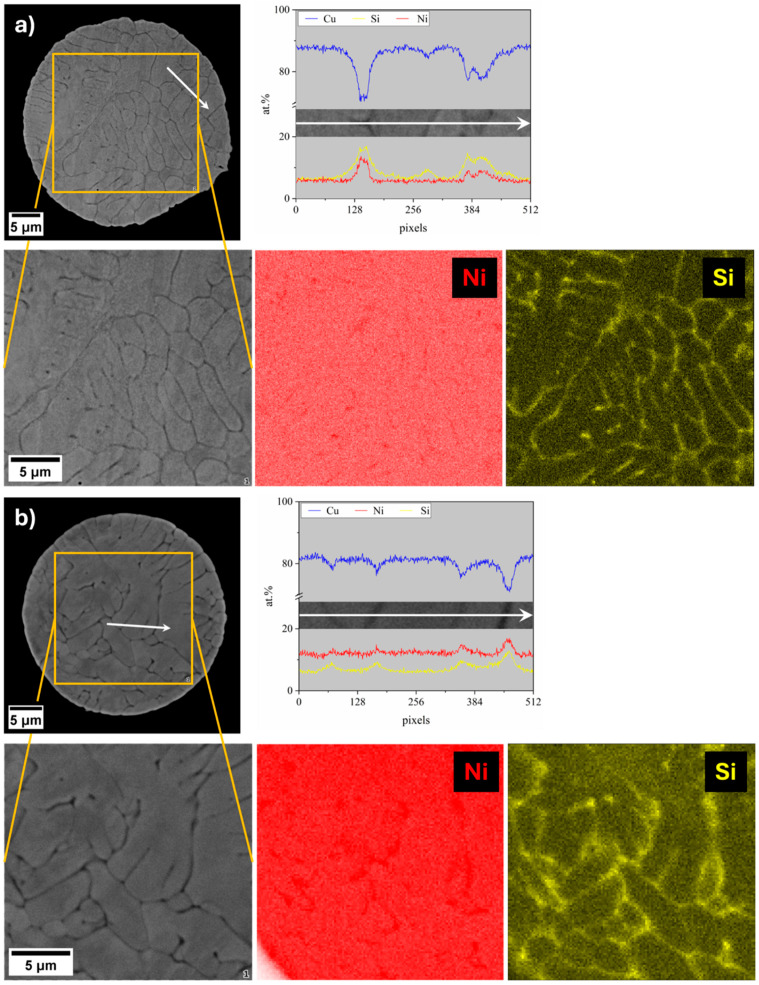
SEM-EDX analyses of (**a**) CuNi3Si1.5 and (**b**) CuNi10Si1.5 particle cross-sections (white arrow marks the line scan path.

As dendritic solidification typically promotes solute partitioning during solidification, SEM-EDX analyses were performed on particle cross-sections. The results, shown in Figure 3, effectively reveal the microsegregation of the alloying elements, leading to the formation of a continuous network-like morphology. While the EDX maps primarily reveal a pronounced microsegregation of Si in both gas-atomized systems, Ni appears more evenly distributed within the matrix. However, line scan analyses highlight the localized Ni and Si enrichment in the interdendritic regions.

**Figure 4:**
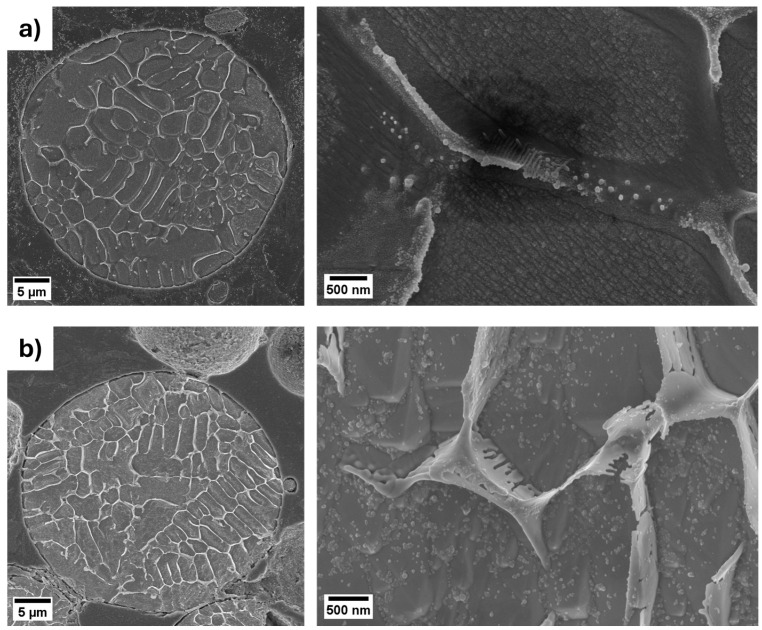
FESEM micrographs of etched (**a**) CuNi3Si1.5 and (**b**) CuNi10Si1.5 particle cross-sections.

The microstructural heterogeneities revealed by the SEM-EDX analyses are further supported by morphological observations at higher magnification on chemically etched particles (Figure 4), which provide a clearer view of the solidification microstructure of the two gas-atomized systems. In both cases, the chemical etching preferentially etched the matrix, thereby enhancing the contrast of the network-like morphology. Particles of CuNi10Si1.5 (Figure 4b) appear to exhibit a slightly thicker and more continuous network structure compared to those of CuNi3Si1.5 (Figure 4a). Notably, similar network structures have been well documented in Cu-Ni-Si cast alloys. Xie et al. proposed a solidification process that leads to the formation of such a microstructure [33]. On the basis of their experimental results, the α-Cu phase nucleates first. As the solidification proceeds, the remaining liquid becomes enriched in Ni and Si and, therefore, approaches a eutectic composition. This leads to the formation of a network of Ni-Si-rich phases at the boundaries of the α-Cu phase. Unlike with casting [34], however, the network structure here observed appears significantly finer, with nanometric dimensions, indicating that the higher cooling rate provided by gas-atomization—far exceeding that of traditional casting [30]—significantly affects this undesired microstructure.

**Figure 5:**
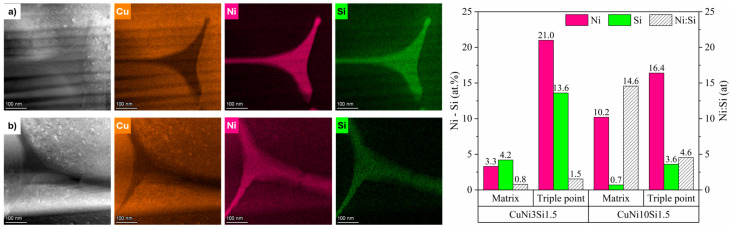
HR-STEM of the two gas-atomized powders: EDX elemental maps at triple points of (**a**) CuNi3Si1.5 and (**b**) CuNi10Si1.5; Ni and Si atomic concentrations, along with Ni:Si ratios, in the matrix and triple-point regions.

To gain deeper insights into the compositions of the observed solidification structures, STEM-EDX analyses were performed. Figure 5 illustrates the local elemental compositions of the microsegregation areas, referred to as triple points, and of the surrounding matrix regions for the two gas-atomized powders. CuNi10Si1.5 particles show triple points particularly enriched in Ni, with a Ni:Si atomic ratio of 4.6, significantly higher than the Ni:Si atomic ratio of 1.5 calculated for the CuNi3Si1.5 particles. With regard to the matrix regions, while the local composition of the CuNi10Si1.5 particles closely reflects the nominal one (Table 1), those of CuNi3Si1.5 exhibit a higher Si content. These findings suggest the critical role of Ni in driving the extent and nature of Ni-Si phase formation. In particular, the lower Ni content in the CuNi3Si1.5 system may limit the formation of Ni-Si precipitates, resulting in a greater retention of Si in solid solution. This is supported by the XRD pattern shown in Figure 1, which revealed a higher fraction of Ni-Si precipitates in the CuNi10Si1.5 powders, as evidenced by the presence of additional peaks with relatively higher intensities.

**Figure 6:**
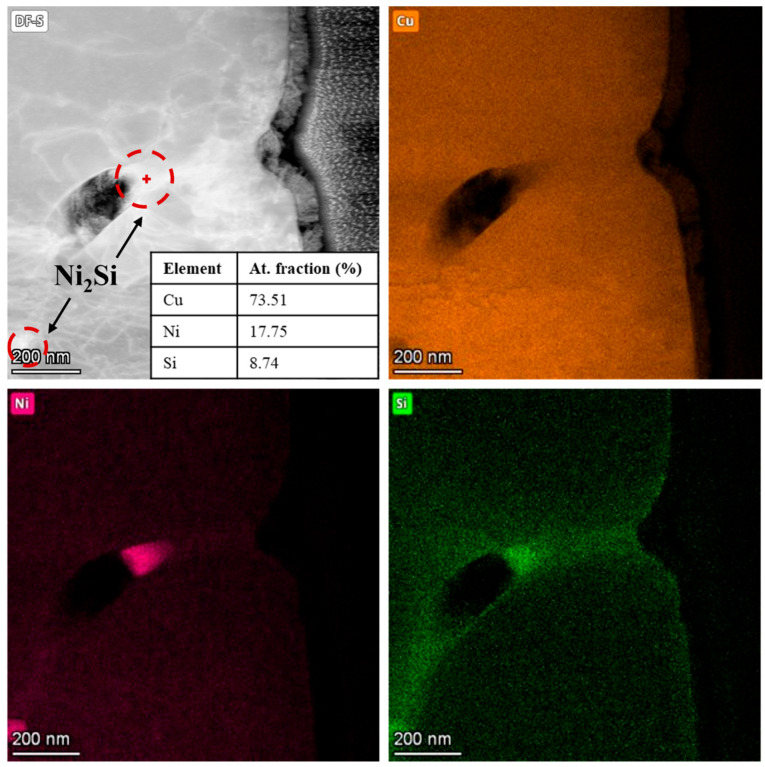
HR-STEM/EDX elemental maps of the CuNi3Si1.5 lamella.

To directly observe and characterize these Ni-Si precipitates, further investigations were carried out using TEM. The dark-field (DF) TEM image of the CuNi3Si1.5 particle depicted in Figure 6, accompanied by the EDX maps, reveals the presence of nanoscale precipitates rich in Ni and Si within the network. The point EDX analysis shows a Ni:Si atomic ratio of approximately of 2:1, thus confirming the presence of Ni_2_Si precipitates, which is in agreement with the reflections observed in the diffraction pattern of the CuNi3Si1.5 powders reported in Figure 1.

**Figure 7:**
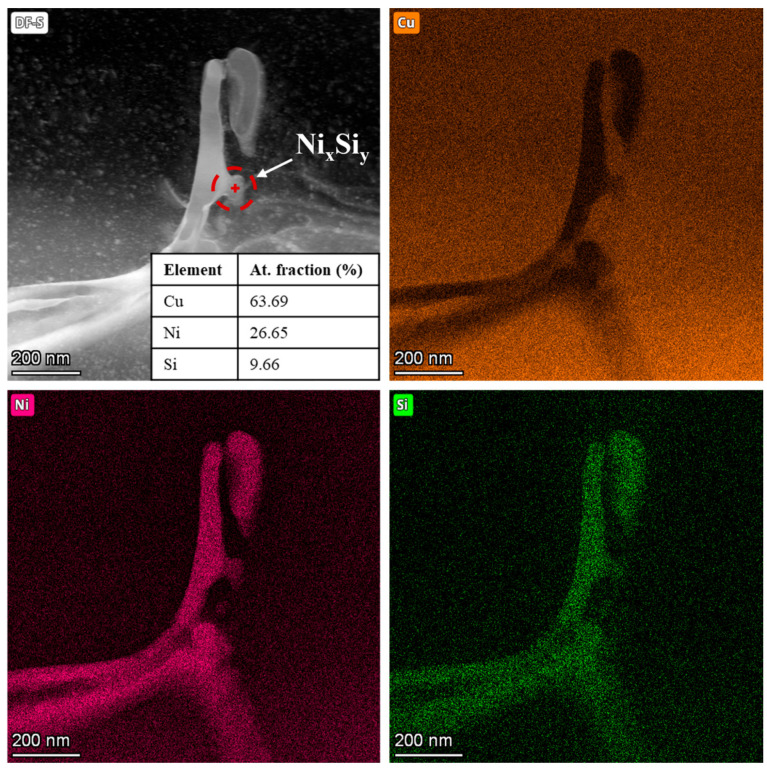
HR-STEM/EDX elemental maps of the CuNi10Si1.5 lamella.

Similarly, the TEM DF image of the CuNi10Si1.5 lamella shown in Figure 7, along with the corresponding EDX maps, reveals the presence of Ni- and Si-rich nanoscale precipitates within the network. However, in this case, the EDX point analysis indicates a Ni:Si atomic ratio of approximately 2.8:1, suggesting that the additional Ni_x_Si_y_ detected by XRD (Figure 1) may correspond to stoichiometries consistent with Ni_3_Si or Ni_31_Si_12_ silicides.

Nanoindentation tests were performed to correlate the observed microstructural features with the mechanical performance of the two systems under investigation. According to the results summarized in Table 4, the particles of CuNi3Si1.5 exhibited a mean nanohardness of 2.17 ± 0.04 GPa (95% confidence interval) with a standard deviation of 0.38 GPa. On the other hand, the particles of CuNi10Si1.5 revealed a mean nanohardness of 3.06 ± 0.06 GPa (95% confidence interval) with a standard deviation of 0.56 GPa. It can be concluded that the higher Ni content led to a significant increase in nanohardness, on average exceeding 40%. Given that the 95% confidence intervals do not overlap, this difference is considered statistically significant. Upon examining the standard deviations, which reflect the spread of the data, it is evident that the CuNi3Si1.5 particles exhibit less variability in their nanohardness values compared to the CuNi10Si1.5 ones, which show greater spread.

**Figure 8:**
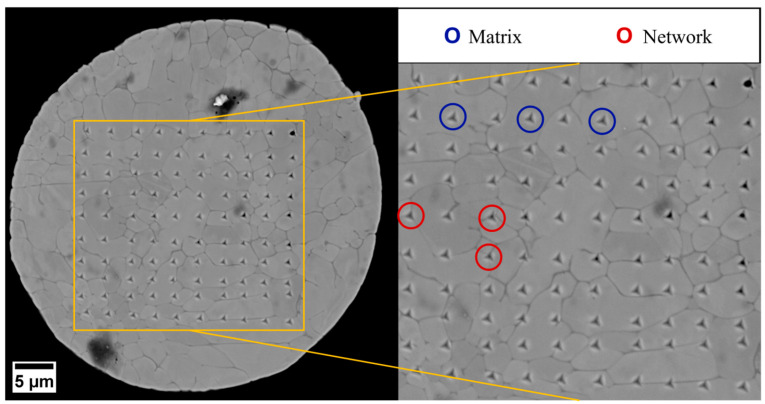
SEM image showing nanoindentation imprints on the particle cross-section: blue circles indicate indentations located in the matrix region, whereas red circles mark those located within the network.

To further investigate the underlying causes of the higher variability observed in the CuNi10Si1.5 system, SEM observations were conducted on the nanoindented particles, as shown in Figure 8 as an example. A distinction was made between indentations located in the matrix regions (blue circles) and those situated in the Ni- and Si-rich interdendritic network (red circles). Due to the scale of the indentations relative to the characteristic microstructural features of the two compositions, it was not possible to unambiguously assign all data points to either the matrix or the network. Therefore, only data from nanoindentations that could be clearly associated with these regions were considered for analysis, ensuring more reliable and robust results. Based on this classification, the CuNi3Si1.5 particles exhibited a nanohardness of 2.10 ± 0.07 GPa for the matrix and 2.24 ± 0.05 GPa for the network, indicating only a minimal difference, as reported in Table 4. On the other hand, the CuNi10Si1.5 particles displayed significantly higher nanohardness values, as follows: 2.83 ± 0.08 GPa in the matrix and 3.33 ± 0.09 GPa in the network (see Table 4). This result is, thus, a direct effect of the solid solution strengthening and the complex precipitate network. The higher Ni content in CuNi10Si1.5 results in a matrix nanohardness increase of nearly 35% compared to CuNi3Si1.5, which can reasonably be attributed to a more pronounced solid solution, as suggested by the lower lattice parameter calculated from the XRD analyses. Similarly, in the CuNi10Si1.5 system, the network exhibits an even greater hardness increase, approaching 50% with respect to the CuNi3Si1.5 results, likely due to a higher volume fraction of Ni-Si precipitates with varied stoichiometries, as indicated by the relatively higher intensity of secondary peaks in the diffraction pattern of this composition (Figure 1b).

**Figure 9:**
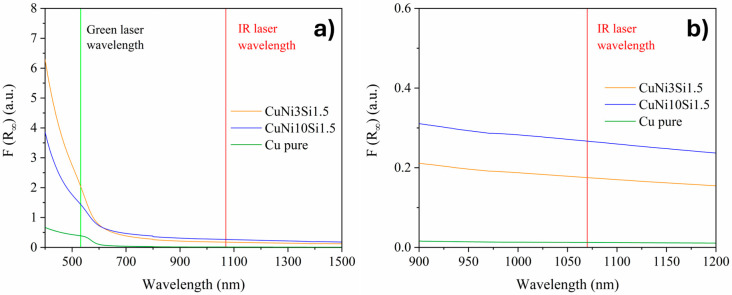
(**a**) UV-Vis spectra of CuNi3Si1.5 (orange line), CuNi10Si1.5 (blue line), and pure Cu (green line) powders; (**b**) magnified view of the wavelength range 900–1200 nm.

In light of their intended application in PBF-LB/M, the optical absorption characteristics of the two gas-atomized systems were finally examined through UV-Vis spectroscopy. As shown in Figure 9, both powders exhibit a significantly enhanced optical response compared to pure Cu across the entire investigated wavelength range. This enhancement can be ascribed to composition-induced modifications of the electronic structure, where Ni and Si in solid solution alter the density of electronic states near the Fermi level and increase electron scattering, thereby reducing reflectivity. In particular, studies on Cu-Ni alloys have shown that Ni additions generate virtual energy states rather than a simple Fermi-level shift, thus modifying optical absorption behavior [35]. Although the observed increase at infrared (IR) wavelengths is moderate compared to the much larger absorptivity gains achievable at shorter, green wavelengths, it remains highly relevant for IR-based PBF-LB/M systems, which are still the most widely adopted industrial configuration in metal AM. A closer comparison of the two gas-atomized systems reveals that the higher Ni content in CuNi10Si1.5 leads to an absorbance increase of over 52% at IR wavelengths. This enhanced absorption is particularly promising, as it should imply a more effective laser–powder coupling during PBF-LB/M of high-Ni Cu-Ni-Si compositions, leading to improved processability and the production of dense bulk parts [36].

## 4. Conclusions

This work explored two gas-atomized Cu-Ni-Si powders—CuNi3Si1.5 and CuNi10Si1.5 (wt.%)—specifically developed for PBF-LB/M in order to investigate the effect of an increase in Ni content on the microstructure, phases, and mechanical properties. The powder characterization can be useful for predicting compositional and microstructural aspects of the related materials processed by PBF-LB/M with a more economic and time-saving approach.

The gas-atomization process enabled the production of highly spherical powders, with a low number of defects and chemical compositions close to the nominal ones. Both powders exhibited a dendritic solidification structure with primary α-Cu dendrites surrounded by a Ni- and Si-rich network in the interdendritic regions. However, compared to casting, this network structure appears significantly finer, reflecting the effectiveness of gas-atomization in suppressing this undesired microstructure. Beyond these initial observations, the comparative characterization of the two gas-atomized powders yielded the following key results:

- XRD analyses revealed a greater lattice parameter contraction for the CuNi10Si1.5 powders, suggesting a stronger solid solution strengthening effect. In addition to the Ni_2_Si diffraction peaks observed for both compositions, CuNi10Si1.5 powder exhibited additional Ni_x_Si_y_ diffraction peaks with slightly higher intensities, consistent with a greater overall fraction of precipitates.
- STEM-EDX confirmed the presence of nanometric Ni-Si precipitates in the interdendritic network of both systems. CuNi3Si1.5 showed a Ni:Si atomic ratio consistent with Ni_2_Si precipitates, while CuNi10Si1.5 exhibited a local Ni:Si atomic ratio of ~2.8:1, suggesting that the additional XRD peaks likely correspond to Ni_3_Si or Ni_31_Si_12_ precipitates.
- Nanoindentation tests revealed significantly higher hardness values for the CuNi10Si1.5 particles in both the α-Cu matrix (nearly 35%) and in the interdendritic network region (approaching 50% higher), which is in agreement with XRD indications of a stronger solid solution and greater fraction (and diversity) of Ni-Si precipitates.
- UV-Vis spectroscopy demonstrated an improved optical response for the two gas-atomized compositions compared to pure Cu, with the CuNi10Si1.5 powders exhibiting a significantly higher absorbance (over 52%) than CuNi3Si1.5 at IR wavelengths relevant for PBF-LB/M.

Overall, these findings suggest that the higher Ni content—and consequently higher Ni:Si ratio—of the CuNi10Si1.5 composition promotes enhanced solid solution strengthening, a greater fraction, and variety of precipitates, leading to higher hardness and potentially greater strength, along with improved optical properties. These features support the potential of high-Ni Cu-Ni-Si formulations for use in PBF-LB/M applications aimed at achieving dense bulk materials with improved final properties.

Finally, these findings offer a new pathway for designing new compositions for AM based on a powder-level characterization, enabling a faster screening of alloy candidates prior to full PBF-LB/M processing.

## Figures and Tables

**Table 1:** Nominal compositions (wt.% and at.%) and weight (kg) of each raw element for the two gas-atomized Cu-Ni-Si systems.

		Nominal Composition			Raw Materials (kg)		
		Cu	Ni	Si	Cu	Ni	Si
CuNi3Si1.5	(wt.%)	Bal.	3	1.5	7.64	0.24	0.12
	(at.%)	Bal.	2.80	0.67			
CuNi10Si1.5	(wt.%)	Bal.	10	1.5	7.08	0.80	0.12
	(at.%)	Bal.	9.34	0.67			

**Table 2:** Chemical compositions (ICP-OES) and light element contents (LECO-ONH) of the two gas-atomized CuNiSi powders.

	Chemical Composition			Light Element Content		
Element (wt.%)	Cu	Ni	Si	O	N	H (ppm)
CuNi3Si1.5	Bal.	2.94	1.37	0.013 ± 0.002	0.028 ± 0.004	1.34 ± 0.19
CuNi10Si1.5	Bal.	9.70	1.36	0.019 ± 0.002	0.041 ± 0.002	1.38 ± 0.29

**Table 3:** D10, D50, and D90 number-based percentiles of the PSD, aspect ratio, and circularity of the two gas-atomized CuNiSi powders.

	CuNi3Si1.5			CuNi10Si1.5		
	D[n,0.1]	D[n,0.5]	D[n,0.9]	D[n,0.1]	D[n,0.5]	D[n,0.9]
PSD (μm)	14.62	23.23	37.15	13.55	20.78	31.30
Circularity	0.913	0.980	0.988	0.909	0.975	0.985
Aspect Ratio	0.800	0.974	0.997	0.788	0.971	0.996

**Table 4:** Measured nanohardness values (mean, matrix, and network) of the two gas-atomized Cu-Ni-Si powders and differences in nanohardness values (Δ*H*).

Alloy		Mean	Matrix	Network
CuNi3Si1.5	*H* (GPa)	2.17	2.10	2.24
	SD (GPa)	0.38	0.25	0.17
	CI_95_ (GPa)	±0.04	±0.07	±0.05
CuNi10Si1.5	*H* (GPa)	3.06	2.83	3.33
	SD (GPa)	0.56	0.20	0.27
	CI_95_ (GPa)	±0.06	±0.08	±0.09
ΔH		+41.0%	+34.7%	+48.7%

*H*—nanohardness; SD—standard deviation; CI_95_—confidence interval.

## Data Availability

The data presented in this study are available on request from the corresponding author. The data are not publicly available due to privacy.
